# METTL16 promotes cell proliferation by up‐regulating cyclin D1 expression in gastric cancer

**DOI:** 10.1111/jcmm.16664

**Published:** 2021-06-02

**Authors:** Xiao‐Kun Wang, Ya‐Wei Zhang, Chun‐Ming Wang, Bo Li, Tian‐Zhi Zhang, Wen‐Jie Zhou, Lyu‐jia Cheng, Ming‐Yu Huo, Chang‐Hua Zhang, Yu‐Long He

**Affiliations:** ^1^ Digestive Disease Center Seventh Affiliated Hospital Sun Yat‐sen University Shenzhen China; ^2^ Gastrointestinal Surgery Center First Affiliated Hospital Sun Yat‐Sen University Guangzhou China; ^3^ The Emergency Department Jingmen First People's Hospital Jingmen China; ^4^ Pathology Department Seventh Affiliated Hospital Sun Yat‐sen University Shenzhen China

**Keywords:** gastric cancer, m6A, METTL16, cyclin D1

## Abstract

N6‐methyladenosine (m6A) is a well‐known modification of RNA. However, as a key m6A methyltransferase, METTL16 has not been thoroughly studied in gastric cancer (GC). Here, the biological role of METTL16 in GC and its underlying mechanism was studied. Immunohistochemistry was used to detect the expression of METTL16 and relationship between METTL16 level and prognosis of GC was analysed. CCK8, colony formation assay, EdU assay and xenograft mouse model were used to study the effect of METTL16. Regulatory mechanism of METTL16 in the progression of GC was studied through flow cytometry analysis, RNA degradation assay, methyltransferase inhibition assay, RT‐qPCR and Western blotting. METTL16 was highly expressed in GC cells and tissues and was associated with prognosis. In vitro and in vivo experiments confirmed that METTL16 promoted proliferation of GC cells and tumour growth. Furthermore, down‐regulation of METTL16 inhibited proliferation by G1/S blocking. Significantly, we identified cyclin D1 as a downstream effector of METTL16. Knock‐down METTL16 decreased the overall level of m6A and the stability of cyclin D1 mRNA in GC cells. Meanwhile, inhibition of methyltransferase activity reduced the level of cyclin D1. METTL16‐mediated m6A methylation promotes proliferation of GC cells through enhancing cyclin D1 expression.

## 1 BACKGROUND

Gastric cancer (GC) is a global health issue, with more than 1 million new cases worldwide every year. Although the incidence and mortality of GC have declined globally in the last 50 years, it currently remains as the third leading cause of cancer‐related deaths with the fifth highest incidence in all of cancer.^1^ Currently, surgery is the only possible radical cure. However, most patients lost the opportunity for surgical treatment due to the fact that they were diagnosed in the mid to advanced stages. Although some progress has been made in the pathogenesis and treatment targets of GC, it is not enough to meet the clinical demands for improving the diagnosis and treatment of GC.^2^ Therefore, exploring the pathogenesis of GC, finding better treatment targets and optimizing treatment strategies are issues that we urgently need to resolve.

As the most abundant internal modification in eukaryotic mRNA, N6‐methyladenosine (m6A) modification affects the splicing, transcription, translation, localization, metabolism and stability of RNA.^3^ m6A modification plays key roles in a multitude of biological processes, such as the development of nervous system, circadian rhythm, DNA damage response, heat shock response, cell signal transduction and tumorigenesis.^4^ There is growing evidence that RNA modification pathways function in the regulation of human cancers and they may be ideal targets for cancer treatment.^5^ Changes of m6A levels in malignant tumours may play a role in promoting or suppressing cancer development through affecting related tumour markers, for example maintaining proliferation signals, evading growth inhibitors, resisting apoptosis, making replicates immortal, inducing angiogenesis, activating invasion and metastasis, reprogramming energy metabolism, promoting genome instability and mutation, inducing evasion of immune surveillance and cancer‐promoting inflammation.^6^, ^7^, ^8^ METTL16 is the second RNA m6A methyltransferase discovered so far, which can modify certain coding and non‐coding RNAs with m6A.^9^ It can regulate MAT2A mRNA level in cell to maintain the steady state of S‐adenosylmethionine (SAM),^10^, ^11^ and it is also indispensable for mouse development.^12^ However, the specific role of METTL16 in the development of cancer, especially GC, and related regulatory mechanism are still unclear.

In our study, we investigated the expression of METTL16 in GC and found that the proliferation of GC cells was significantly inhibited after knocking down METTL16, and the cell cycle was blocked in G1/S phase. By search target of this effect, we found cycle D1 as a key downstream target for this RNA modification. Our results suggested that METTL16 might be a potential therapeutic target for the treatment of human GC.

## 2 METHODS AND MATERIALS

### 2.1 Clinical specimens and ethical approval

We had collected 231 paraffin‐processed GC samples with its paired normal adjacent tissues (NATs) from patients who underwent radical GC surgery from January 2008 to December 2013 in the First Affiliated Hospital of Sun Yat‐sen University. The 5 µm paraffin sections were completed in the pathology department, along with complete follow‐up data provided by the GI surgical department of the hospital. Follow‐up period was once every 3 months in the first 2 years, and once every 6 months from the 3rd to 5th years, with a mean follow‐up period of 49.1 months. Total survival time was defined as from the day of surgery to the time of death or date of last follow‐up. Clinicopathological characteristics of the patients can be found in Table 1. We have also collected 16 fresh GC samples with its paired NATs from patients who underwent radical GC surgery from June 2019 to September 2019 in the Seventh Affiliated Hospital of Sun Yat‐sen University, including 10 male cases and 6 female cases, with a median age of 59 years (47‐72 years). These fresh samples underwent rapid freezing with liquid nitrogen upon excision and then stored in −80°C refrigerator until use. Research protocol was approved by both Ethics Committee of the First and Seventh Affiliated Hospital of Sun Yat‐sen University.

**TABLE 1:** Associations of METTL16 expression with clinical parameters in gastric cancer

Characteristic	No.	METTL16 expression		*P* value
		Low(N = 115)	High(N = 116)	
Age(year)		55.8 ± 11.9	58.3 ± 12.4	0.934
≤60y	142	71	71	
>60y	89	44	45	
Gender				0.544
Male	155	75	80	
Female	76	40	36	
Tumour location				0.716
Upper	60	30	30	
Other	171	85	86	
Tumour size				**0.004**
<5 cm	128	75	53	
≥5 cm	103	40	63	
Differentiation				0.631
Well + Moderate	65	34	31	
poor	166	81	85	
Depth of invasion				0.051
T1 + T2	59	36	23	
T3 + T4	172	79	93	
Lymph node metastasis				**0.043**
N0	74	44	30	
N+	157	71	86	
Distant metastasis				0.568
Yes	29	13	16	
No	202	102	100	
CEA level μg/L)				0.762
<5	187	94	93	
≥5	44	21	23	
Vessel or nerve invasion				0.843
Yes	45	23	22	
No	186	92	94	

Bold indicates statistical significant value.

### 2.2 Immunohistochemistry (IHC)

We employed immunohistochemistry to detect expression levels of METTL16 in GC tissues and its paired NATs. Firstly, the sections were deparaffinized and rehydrated. 1X Tris‐EDTA buffer was used for antigen retrieval at 100°C for 8 minutes, and then, the sections were treated with 3% hydrogen peroxide for 20 minutes, followed by 5% goat serum for 30 minutes. Rabbit anti‐METTL16 polyclonal antibody (1:200; cat. no. A118157; SIGMA) was used overnight at 4°C. Subsequently, after washing with PBS, the section was incubated for 1h at room temperature with horseradish peroxidase (HRP) (goat anti‐rabbit, cat. no. A0208; Beyotime Institute of Biotechnology). After DAB staining and haematoxylin staining, METTL16 expressions in the tissues were observed under a light microscope. An improved H scoring system was used to semi‐quantitate the expression of METTL16. Formula: maximum staining intensity (0, negative ‐; 1, weak positive +; 2, moderately positive++; 3, strong positive +++) multiplied by the percentage of positive tumour cells (0%‐100%) equal corrected H score (range 0‐300).^13^ The immunohistochemical score was scored independently by two pathologists following the scoring method above. They were blinded to the clinical information of the patient, and the final H score was averaged. METTL16 staining was classified as high or low expression according to the median H score.

### 2.3 Immunofluorescence (IF) staining

Fresh mouse subcutaneous tumour tissue was fixed in 4% paraformaldehyde for 24 hours before dehydration, embedded in paraffin and made into 5um sections. The sections were left overnight at 4°C with rabbit anti‐METTL16 polyclonal antibody (1:100; cat. no. A118157; SIGMA) and anti‐Cyclin D1 (1:100; Proteintech, cat#60186‐1‐Ig). Secondary antibodies conjugated with the following fluorescent dyes were used: Alexa Fluor 546–conjugated donkey anti‐rabbit IgG (1:200; cat. no. A11003) and Alexa Fluor 488–conjugated donkey anti‐mouse IgG (1:200; cat. no. A11001). The sections were lastly counterstained with DAPI nucleic acid stain (Invitrogen Molecular Probes, Catalogue number D1306). Images were collected by Leica TCS SP2 confocal system (Leica Microsystems) through Leica's confocal software.

### 2.4 Cell lines and cell culture

One gastric epithelial cell line (GES‐1) and six GC cell lines (AGS, MGC803, SNU719, HGC27, SGC7901 and MKN28) were selected from Chinese Academy of Sciences (Shanghai). AGS cells were cultured in DMEM/F12 (BI, CAT#01‐172‐1ACS) + 10% foetal bovine serum (Cellmax, CAT#SA102.02). MGC803, SNU719, HGC27, SGC7901 and MKN28 cells were cultured in RPMI‐1640 (BI, CAT#01‐100‐1ACS) containing 10% foetal bovine serum. The culture environment was maintained at 37°C and 5% CO_2_ concentration.

### 2.5 METTL16‐targeting short hairpin RNA (shRNA) and lentivirus packaging

Three targeted shRNA and 1 non‐targeted scrambled RNA sequence were sub‐cloned into GV493 lentiviral vector by Shanghai GeneChem Co., Ltd. The target sequence of shMETTL16 is 5'‐AGGGAGTAAACTCACGAAATCCT −3'(shMETTL16‐1), 5'‐CCCTTGAGACTCAACTATATT −3' (shMETTL16‐2) and 5'‐ATGGCTGGTATTTCCTCGCAA‐3'(shMETTL16‐3); the non‐targeting disruptive RNA sequence is 5'‐TTCTCCGAACGTGTCACGT‐3'(shNC). In addition, the target sequence of METTL16 overexpression is 5'‐CGCAAATGGGCGGTAGGCGTG‐3'(OE). The lentivirus strains were prepared and purified following the manufacturer's kit instructions.

### 2.6 EdU cell proliferation assay

METTL16 gene knock‐down GC cells and their corresponding control cells were seeded in a 24‐well culture plate at a rate of 2 × 10^5^ cells/well and incubated for 24 hours. The EdU analysis kit (Beyotime Biotechnology, CAT#C0075S) was used to analyse and evaluate cell proliferation, and the specific method was carried out following the instructions provided by the manufacturer. The samples were analysed with a Leica fluorescence microscope.

### 2.7 Cell cycle analysis

The treated cells were collected and fixed with 75% pre‐chilled ethanol at 4°C overnight. After removing the ethanol, phosphate buffer was used to wash the cells twice, and then, the cells are incubated with propidium iodide staining solution in the cell cycle analysis kit (Beyotime, CAT#C1052) for 30min at room temperature. Cell cycle distribution was analysed through flow cytometry (Beckman CytoFlex).

### 2.8 Real‐time quantitative PCR analysis

Following the manufacturer's instructions, AG RNAex Pro RNA reagent (Accurate Biology, CAT#AG21102) was used to extract total RNA from tissue samples or cell lines. Evo M‐MLV reverse transcription master mix (Accurate Biology, CAT#AG11706) was used to synthesize cDNA from 2 μg RNA of each sample. SYBR Green Pro Taq HS premixed qPCR kit (Accurate Biology, CAT# AG11701) was used for qRT‐PCR. METTL16 primer sequence: forward, 5'‐CTCTGACGTGTACTCTCCTAAGG‐3' and reverse, 5'‐TACCAGCCATTCAAGGTTGCT‐3'. GAPDH: forward, 5'‐GGAGCGAGATCCCTCCAAAAT‐3' and reverse, 5'‐GGCTGTTGTCATACTTCTCATGG‐3'. CDK2: forward, 5'‐CCAGGAGTTACTTCTATGCCTGA‐3' and reverse, 5'‐TTCATCCAGGGGAGGTACAAC‐3'. CDK6: forward, 5'‐GCTGACCAGCAGTACGAATG‐3'and reverse, 5'‐GCACACATCAAACAACCTGACC‐3'. cyclin D1: forward, 5'‐GCTGCGAAGTGGAAACCATC‐3' and reverse, 5'‐CCTCCTTCTGCACACATTTGAA‐3'. cyclin E1: forward, 5'‐AAGGAGCGGGACACCATGA‐3' and reverse, 5'‐ACGGTCACGTTTGCCTTCC‐3'. Each sample had 3 repeated tests. Data were analysed via the 2^−ΔΔCT^ calculation method.

### 2.9 CCK8 cell proliferation assay and colony formation assay

In the cell proliferation assay, 2 × 10^3^ cells/well were seeded into 96‐well plates. After the cells adhered, 10 ul of CCK8 reagent (Fude Biological, CAT#FD3788) was added to each well on day 1, 2, 3, 4 and 5, and the absorbance was measured by spectrophotometry at 450 nm wavelength after 2 hours.

In the colony formation assay, 500 cells/well were seeded in a 6‐well culture dish. After 2 weeks, the cells were fixed in 4% paraformaldehyde, stained with crystal violet (Beyotime Biotechnology, CAT#C0121) and counted microscopically.

### 2.10 Western blotting and antibodies

Cells were collected and placed into a protein lysis buffer on ice for 30 minutes. BCA protein assay kit (KeyGEN BioTECH, CAT#KGP903) was used to quantify protein concentration. The proteins were separated by 10% SDS‐polyacrylamide gel electrophoresis (SDS‐PAGE). Next, the sample was transferred to a polyvinylidene difluoride (PVDF) membrane with pore size of 0.45 μm (Merck Millipore, CAT# IPVH00010). The non‐specific binding sites on the membrane were blocked with 5% bovine serum albumin (Beyotime Biotechnology, CAT# ST023‐1000g) for 1h. After blocking, the membrane was first incubated with the primary antibody overnight at 4°C and then with the secondary antibody. Finally, super‐sensitive ECL assay kit was used (Beyotime Biotechnology, CAT# P0018AM) to show the immune response, and two‐colour infrared fluorescence imaging system (Bio‐Rad ChemiDoc MP) was used to image the spots. Following antibodies were used: anti‐METTL16 (SIGMA, HPA020352), anti‐Cdk2 (Abcam, cat#ab32147), anti‐Cdk6 (Abcam, cat#ab124821), anti‐cyclin D1 (Proteintech, cat#60186‐1‐Ig), anti‐cyclin E1 (Proteintech, cat#11554‐1‐AP), anti‐p21 (Proteintech, cat# 10355‐1‐AP) and anti‐GAPDH (Proteintech, cat#60004‐1‐Ig).

### 2.11 Quantification of total m6A RNA

We used m6A RNA Methylation Quantification Kit (Colorimetric) to measure the total m6A RNA level in GC cells. EpiQuik m6A RNA Methylation Quantification Kit (EpiGentek, cat# P‐9005) is a complete set of optimized buffers and reagents for colorimetric quantification of m6A in RNA. It is suitable for directly measuring m6A RNA methylation status from total RNA.^14^ 200 ng of RNA was injected into the test wells, and detection antibody solution of the appropriate dilution concentration was added into the test wells according to the manufacturer's instructions. The m6A level was quantified by colorimetry by measuring the absorbance of each well at 450nm, and the m6A level was calculated according to the standard curve.

### 2.12 RNA decay assay

5 × 10^5^ shNC or shMETTL16 gastric cancer cells were added to each well of a 6‐well plate. After culturing overnight, actinomycin D was added (Mce, HY‐17559) to each well, bringing the final concentration to 5 μg/mL. Cells were collected after 0, 2, 4 and 8h. Total RNA was extracted, and RT‐qPCR was performed to quantify the isotopic abundance ratio of cyclin D1 mRNA (relative to 0h).

### 2.13 Methyltransferase inhibition test

0.5 × 10^5^ gastric cancer cells were added into each well in a 6‐well plate. After the cells adhered, they were treated with 3‐ deazaadenosine DAA (ApexBio Technology, B6121) at the concentration of 0, 100 and 200 µmol/L. After 24 hours, the expression of related molecule mRNA and protein was detected by RT‐qPCR or Western blotting. DAA is a specific inhibitor of methyltransferase, which could inhibit the methyltransferase activity of METTL16.

### 2.14 Animal experiments

Xenograft mouse model was used to verify the tumorigenic effect of METTL16 in vivo. BALB/c nude mice (4 weeks old) were injected with METTL16 gene knock‐down stable MGC803 GC cells (3 × 10^6^ cells/mice, subcutaneous injection) or shNC control cells (3 × 10^6^, subcutaneous injection), and the dose was 100 μl, with PBS as solvent. The tumour size was measured every 3‐5 days. At the end of feeding (6 weeks after subcutaneous injection), the mice were killed and the tumours were extracted for histological analysis. The mass of the tumour was measured, and the following formula was used to estimate the volume: ½ × (length×width^2^). 10 BALB/c nude mice, female, 4 weeks old, were selected from Beijing Vital River Laboratory Animal Technology Co., Ltd. and raised in Ruiye Animal Model Experimental Center. All animal experiments were approved by the Experimental Animal Ethics Committee of Ruiye Animal Model Center.

### 2.15 Statistical analysis

All results were expressed as mean±standard deviation. The differences between categorical variables were tested via χ2 test, and the differences between two groups were tested via Student's *t* test. Kaplan‐Meier curve and log‐rank test were used for statistical analysis of OS other survival‐related data of GC patients. SPSS 17.0 software was used for statistical analysis. *P* value < .05 was considered statistically significant.

## 3 RESULTS

### 3.1 METTL16 is up‐regulated in GC tissues and GC cell lines

**FIGURE 1:**
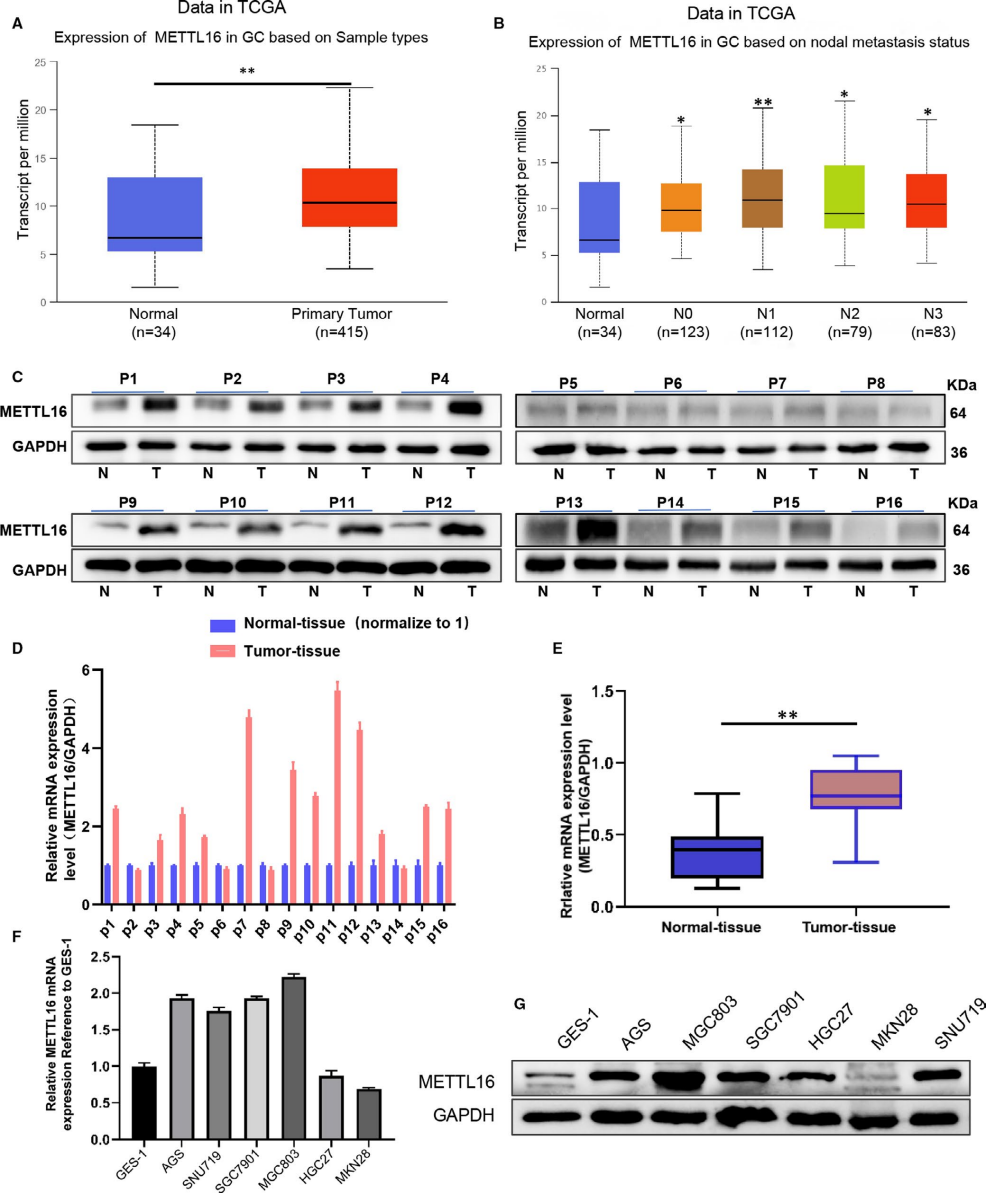
METTL16 is highly expressed in gastric cancer cell lines and tumour tissues. (A) The mRNA level of METTL16 in normal tissues and GC tissues downloaded from The Cancer Genome Atlas (TCGA) database (**P < .01). (B) mRNA level of METTL16 in normal control and different stages (N0‐N4) of GC patients downloaded from the TCGA database (*P < .05,**P < .01 compared with normal tissues). (C) Western blotting was performed to detect the protein level of METTL16 in 16 pairs of GC tissues and their paired normal adjacent tissues. (D and E) The mRNA level of METTL16 in 16 pairs of GC tissues and their paired normal adjacent tissues (D) and the summarized data (E) (**P < .01). (F) mRNA levels of METTL16 in 6 GC cell lines (AGS, SGC7901, SNU719, MGC803, HGC27, MKN28) and 1 normal gastric mucosal cell line (GES‐1). (G) Protein level of METTL16 in 6 GC cell lines and 1 normal gastric mucosal cell line (GES‐1)

In order to investigate whether METTL16 is abnormally expressed in GC, we first compared the expression profiles between GC tissues and the normal adjacent tissues using mRNA expression data set from The Cancer Genome Atlas (TCGA) database. The results showed that the level of METTL16 in GC tissues was significantly higher than that in paired NATs (Figure 1A), and the mRNA level of METTL16 in different stages (N0‐N4) of GC patients was significantly higher than that in normal tissues (Figure 1B). Western blotting and qPCR analysis were then used to detect the expression of METTL16 in 16 cases of GC and its paired NATs. We found that the protein (Figure 1C) and mRNA levels (Figure 1D,E) of METTL16 were higher in most GC tissues compared with the paired NATs. In addition, we detected METTL16 levels in several GC cell lines and found that both mRNA level (Figure 1F) and protein level (Figure 1G) of METTL16 were elevated in most GC cell lines when compared with the normal gastric mucosal epithelial cells GES‐1. The above data indicated that METTL16 level was increased significantly in GC tissues and GC cell lines.

### 3.2 High expression of METTL16 in GC suggests a poor prognosis

**FIGURE 2:**
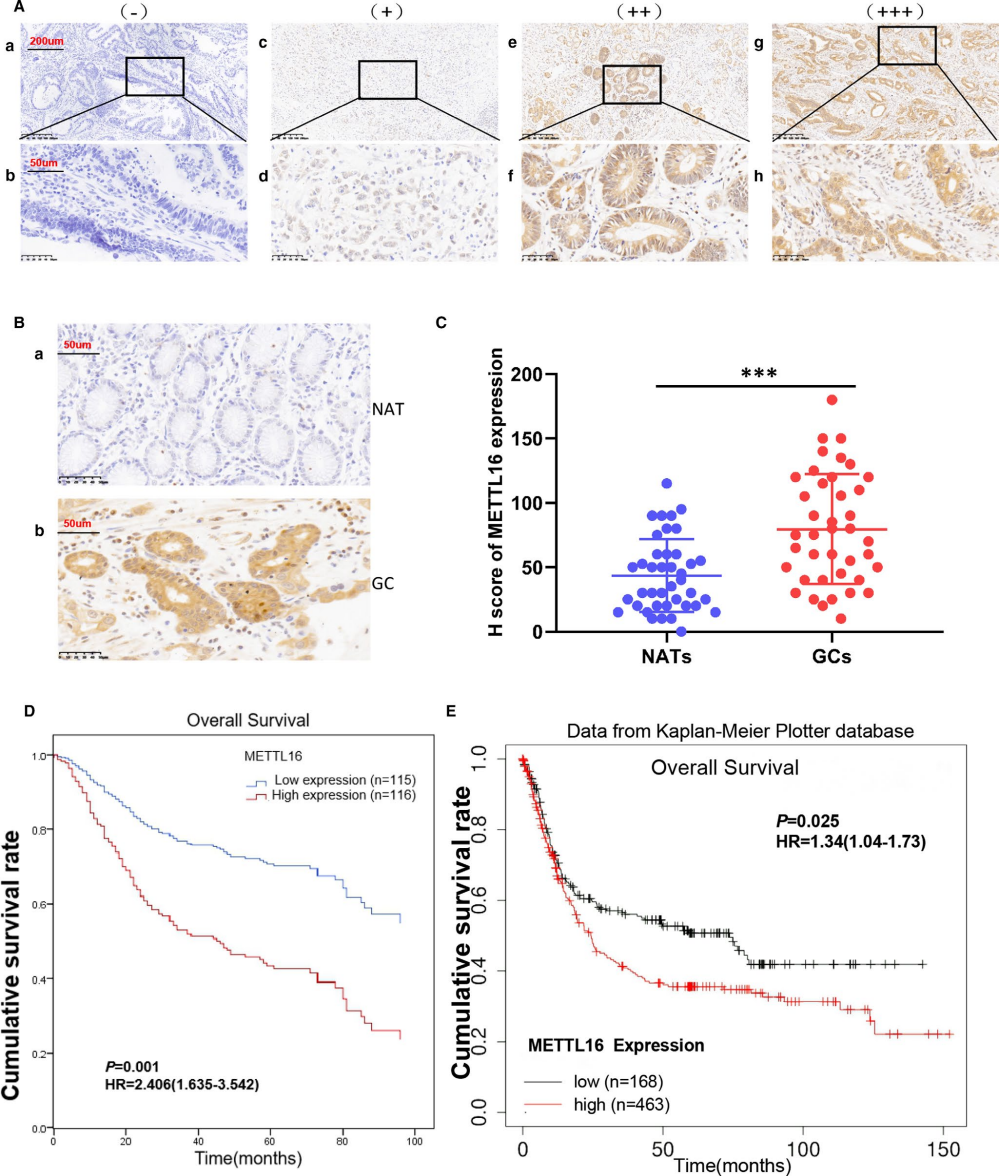
High expression of METTL16 in GC is related to poor survival of patients. (A and B) Immunohistochemical staining of METTL16 in GC tissues and paired normal adjacent tissues. (A) Representative image of GC tissues with negative (A and B), weak positive (C and D), moderately positive (E and F) and strong positive (g and h) METTL16 expression. The images are magnified 100X (A, C, E and G) and 400X (B, D, F and H). (B) Representative image of METTL16 staining in GC tissues and the paired normal adjacent tissues (NAT) (magnified 400X). (C) H score of METTL16 staining in GC tissue and the paired normal adjacent tissues (*n* = 40, H score 43.63 ± 28.41 vs 79.65 ± 42.80,***P ﹤ .001 compared with paired normal adjacent tissues).(D) COX regression model for multivariate analysis showed that the expression level of METTL16 was significantly negatively correlated with the overall survival rate in 231 GC patients (log‐ranch test). (E) The expression level of METTL16 in GC patients was significantly negatively correlated with the overall survival rate in the Kaplan‐Meier plotter database (238931_at)

In order to explore the impact of the high expression of METTL16 on the prognosis of GC patients, immunohistochemistry was used to detect expression level of METTL16 in GC tissues from 231 GC patients with complete follow‐up data. METTL16 was expressed in both cytoplasm and nucleus of cancer cells, and the expression level of METTL16 in gastric cancer cells was much higher than that in surrounding non‐cancer cells (Figure 2A,B). We then used the H score to quantify the staining intensity of METTL16 in these tissues, and the results indicated that the expression of METTL16 in GC tissues was significantly increased compared with the NATs (Figure 2B,C). We also studied the relationship between METTL16 expression and clinicopathological variables in Table 1, and it is worth noting that patients with larger tumour size (≥5 cm) and regional lymph node metastasis tended to show higher level of METTL16 in tumour (Table 1).

Next, we studied the relationship between METTL16 level and the prognosis of GC patients. The results of COX regression model analysis found that patients with higher level of METTL16 had shorter overall survival (OS) compared with those with low level of METTL16 [estimated average OS 60.8 months, 95% confidence interval (CI) 51.8‐69.9 months vs. OS 84.9, 95% CI 74.9 months‐94.9 months; log‐rank test, *P* = .001; Figure 2D]. We further analysed METTL16 transcription data set from Kaplan‐Meier plotter database to verify the relationship between METTL16 level and prognostic effect of GC patients. The results indicated that high expression of METTL16 was associated with poor OS (Figure 2E). The proportional hazards model Cox multivariate analysis also revealed that high level of METTL16 was significantly correlated with lower OS [risk ratio (HR), 1.948; 95%CI, 1.301‐2.915; *P* = .001] (Table 2) after adjusting for tumour size, depth of invasion, lymph node staging, metastasis staging, vascular invasion and other factors. In summary, these findings indicated that METTL16 was positively associated with poor prognosis and it could be used as a prognostic marker for GC.

**TABLE 2:** Cox proportional hazard regression analysis for overall survival

Characteristic	Univariate analysis				Multivariate analysis			
	*P*‐Value	HR	95% CI for Exp(B)		*P*‐Value	HR	95% CI for Exp(B)	
			Lower	Upper			Lower	Upper
Gender								
Male *vs* Female	0.149	1.315	0.906	1.909				
Age								
≤60 y *vs* >60 y	0.885	0.973	0.670	1.412				
Tumour size								
<5cm *vs* ≥5 cm	0.000	2.242	1.551	3.242				
Differentiation								
well+moderate *vs* poor	0.409	1.191	0.786	1.806				
Depth of invasion								
T1/2 *vs* T3/4	0.000	4.325	2.376	7.874	**0.006**	2.442	1.291	4.620
Lymph node metastasis								
N0 *vs* N+	0.000	4.153	2.501	6.896	**0.001**	2.473	1.433	4.268
Distant metastasis								
M0 *vs* M1	0.000	3.224	2.045	5.081	**0.000**	2.352	1.467	3.772
CEA level								
<5 μg/L *vs* ≥5 μg/L	0.111	1.419	0.922	2.184				
Vessel or nerve invasion								
No *vs* yes	0.001	1.972	1.300	2.991				
METTL16 expression								
Low *vs* High	0.000	2.503	1.710	3.663	**0.001**	2.406	1.635	3.542

Bold indicates statistical significant value.

### 3.3 Down regulation of METTL16 inhibits GC cell proliferation through restraining G1/S phase

**FIGURE 3:**
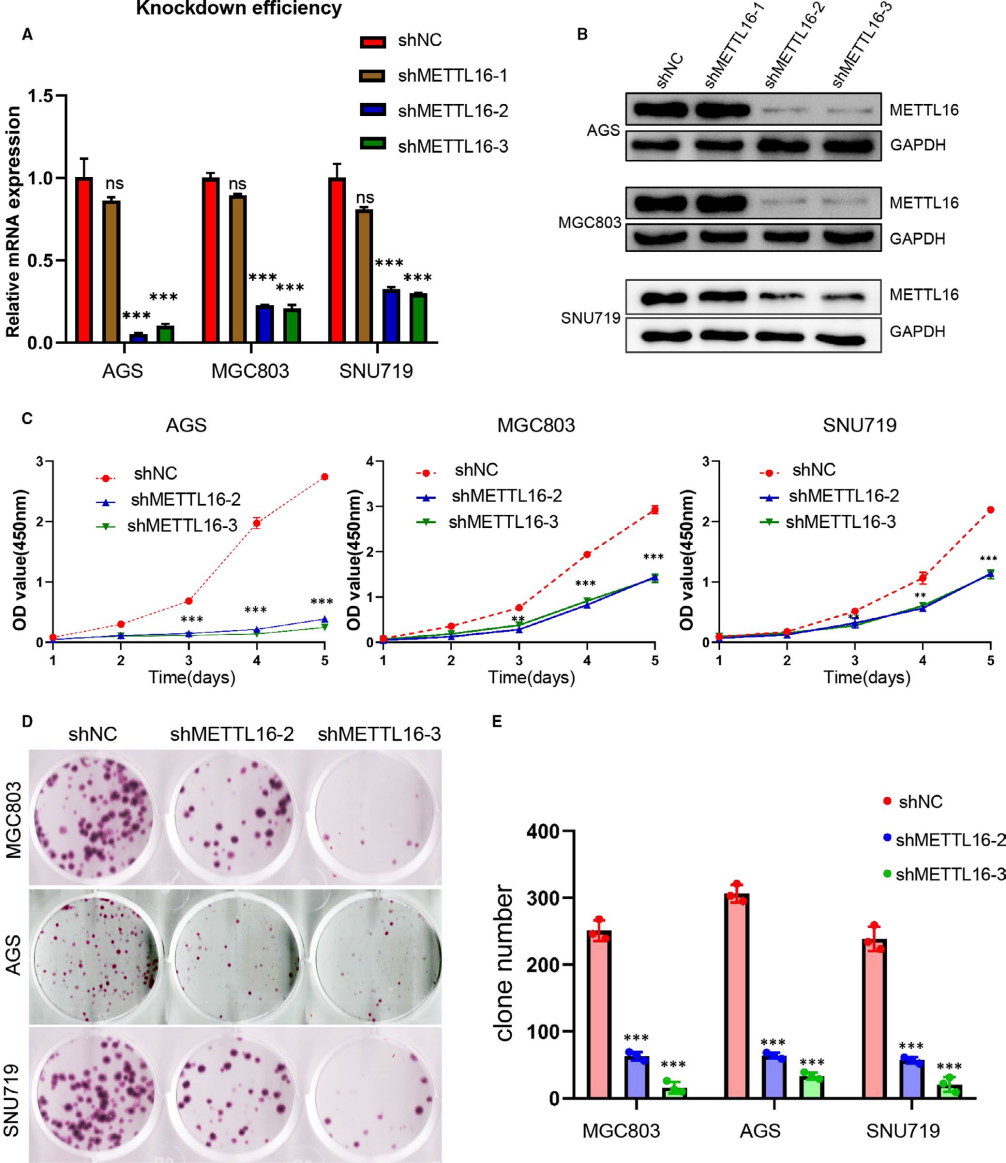
Knock‐down of METTL16 inhibits the proliferation of GC cells in vitro. (A) The mRNA expression of METTL16 in three GC cell lines (AGS, MGC803 and SNU719) after treated with METTL16 shRNA lentivirus. (B) The protein expression of METTL16 in three GC cell lines after treated with METTL16 shRNA lentivirus. (C) Knock‐down of METTL16 can effectively inhibit cell growth in AGS, MGC803 and SNU719 cells. (D and E) Colony formation of AGS, MGC803 and SNU719 cells after transfection of shMETTL16 or shNC. All experiments were performed in triplicate. *P < .05, **P < .01

In order to study the role of METTL16 in GC development, we first verified the METTL16 level in GC cell lines and found that METTL16 expression was significantly increased in most GC cell lines when compared with GES‐1 (Figure 1E,F); thus, we chose AGS, MGC803 and SNU719 three GC cell lines for the next experiments. shRNA was used to knock‐down METTL16 in GC cell lines, and it resulted in higher knock‐down efficiency of shMETTL16‐2 and shMETTL16‐3 than shMETTL16‐1 (Figure 3A,B), so we used shMETTL16‐2 and shMETTL16‐3 in follow‐up experiments. We found that knock‐down of METTL16 inhibited cell growth (Figure 3C) and clonogenic ability (Figure 3D,E) significantly in AGS, MGC803 and SNU719 through CCK8 cell viability assay and colony formation assay, respectively. Overexpression of METTL16 could promote the proliferation of gastric cancer cells (Figure S1A,B) and colony formation (Figure S1C,D). The above results indicated that METTL16 promoted GC cell proliferation.

**FIGURE 4:**
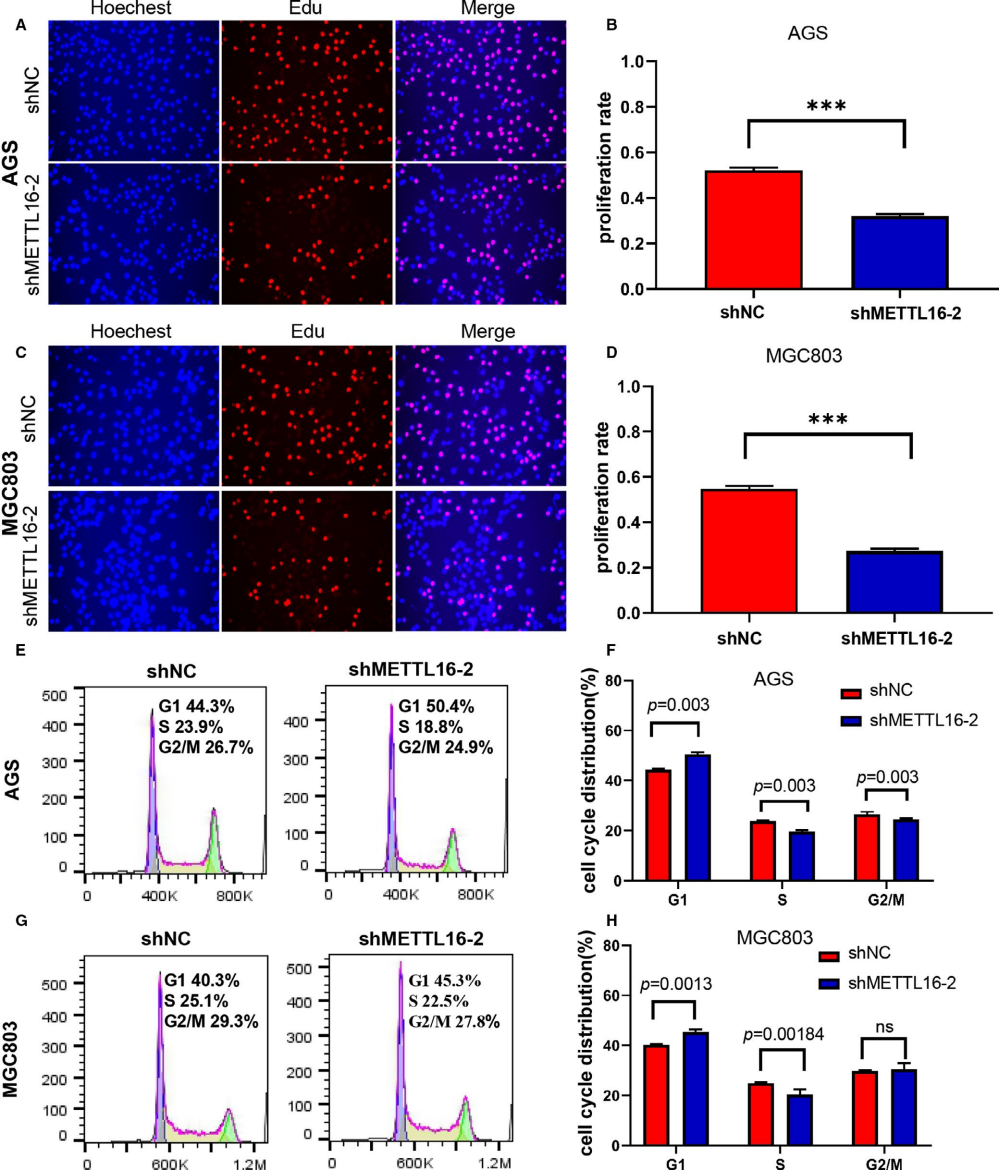
Down‐regulation of METTL16 inhibits the proliferation of GC cells through regulating the cell cycle. (A‐D) Representative immunofluorescence image showing the expression of EdU in AGS (A and B) and MGC803 cells (C and D). (E‐H) Flow cytometry was performed to analyse the cell cycle in shNC‐ or shMETTL16‐treated AGS (E and F) and MGC803 cells (G and H). The knock‐down of METTL16 effectively inhibited the G1/S transition in AGS (E and F) and MGC803 cells (G and H). All results are expressed as the ± SD of three repeated experiments (*0.01 ≤ *P* < .05; **0.001 ≤ *P* < .01, ***P < .001)

To further study the mechanism of METTL16 in promoting the proliferation of GC cells, we conducted an EdU detection test and found that the proportion of S phase cells (DNA synthesis phase) decreased significantly in METTL16 knocked down GC cells (Figure 4A,B,C,D). Additionally, flow cytometry results showed that GC cell cycle was arrested at G1/S phase when METTL16 was knocked down (Figure 4E,F,G,H), while overexpression of METTL16 could promote the G1/S transition in AGS and MKN28 cells (Figure S1E,F,G,H). The above results suggested that METTL16 promotes GC cell proliferation through accelerating the transition of G1/S phase.

### 3.4 METTL16 promotes tumour growth in mice

**FIGURE 5:**
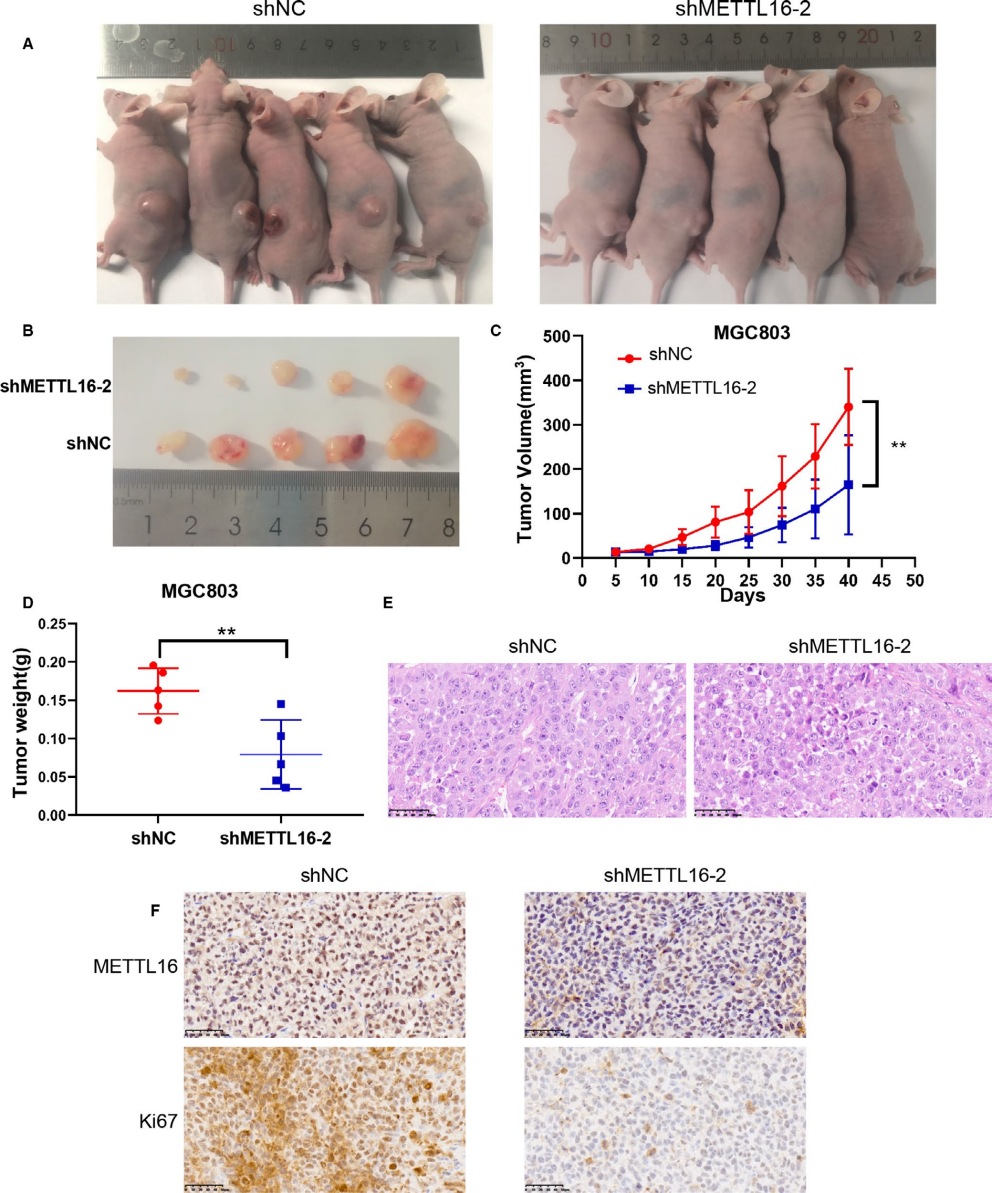
METTL16 promotes tumour growth in vivo. (A) Nude mice are implanted with shNC or shMETTL16‐2 GC cells subcutaneously. (B) Subcutaneous tumour nodules formed in two groups of mice. (C) The growth curve of subcutaneous tumour volume in two groups of mice. (D) Comparison of tumour mass using independent Student's t‐test. (E) HE staining of two groups of tumour specimens. (F) Two groups of tumour specimens were subjected to immunohistochemical detection of METTL16 and Ki67 indicators

To confirm the function of METTL16 in vivo, METTL16 knocked down MGC803 cells (shMETTL16‐2) or normal control MGC803 cells (shNC) were injected subcutaneously into nude mice to establish tumour xenograft models. When compared with control, we found that METTL16 depletion inhibited tumour growth (Figure 5A,B), along with reduced tumour volume and mass (Figure 5C,D). HE staining showed that there were obvious tumour cells in both groups (Figure 5E). The expression of Ki67, a marker of proliferation, also decreased after METTL16 knock‐down (Figure 5F). These results showed that tumour growth is promoted by METTL16 in vivo.

### 3.5 METTL16 regulates the GC cell cycle through mediating the expression of cyclin D1

**FIGURE 6:**
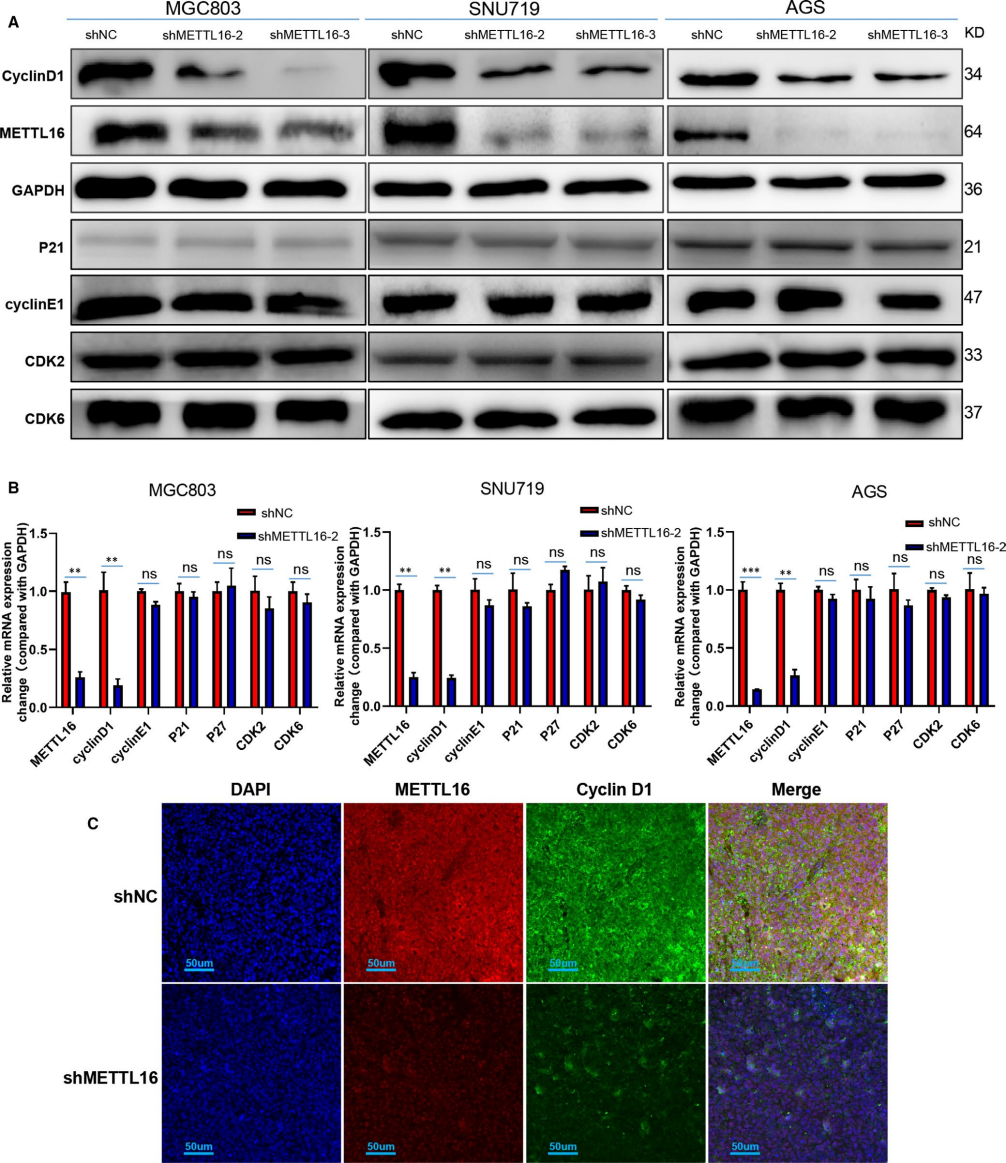
Knock‐down of METTL16 inhibits the expression of cyclin D1 in GC cells. (A) Western blotting analysis was performed to detect the expression of proteins associated with G1/S phase transition, including cyclin D1, cyclin E1, p21, CDK2 and CDK6. (B) qPCR analysis of the expression of cyclin D1, cyclin E1, p21, p27, CDK2 and CDK6. (C) Representative immunofluorescence image showing the expression of cyclin D1 and METTL16 in subcutaneous tumour tissue of mice

In order to clarify the mechanism of the down‐regulation of METTL16 leading to G1‐S phase arrest, we detected the expression of cyclin D1, cyclin E1, p21, p27, CDK2 and CDK6, which are involved in transition of G1/S phase. We found that in METTL16 knocked down AGS, MGC803 and SNU719 cells, the expression of cyclin D1 was significantly reduced (Figure 6A). Similarly, the qPCR results showed that the mRNA level of cyclin D1 was significantly decreased in GC cells after knocking down METTL16 (Figure 6B). In addition, we performed immunofluorescence staining in the tumour tissue sections of xenograft mice and found that cyclin D1 expression was dramatically inhibited in the METTL16 knocked down group (Figure 6C). These results indicated that METTL16 may regulate the cell cycle through mediating the expression of cyclin D1.

### 3.6 METTL16 regulates the expression of cyclin D1 through mediating the stability of cyclin D1 mRNA

**FIGURE 7:**
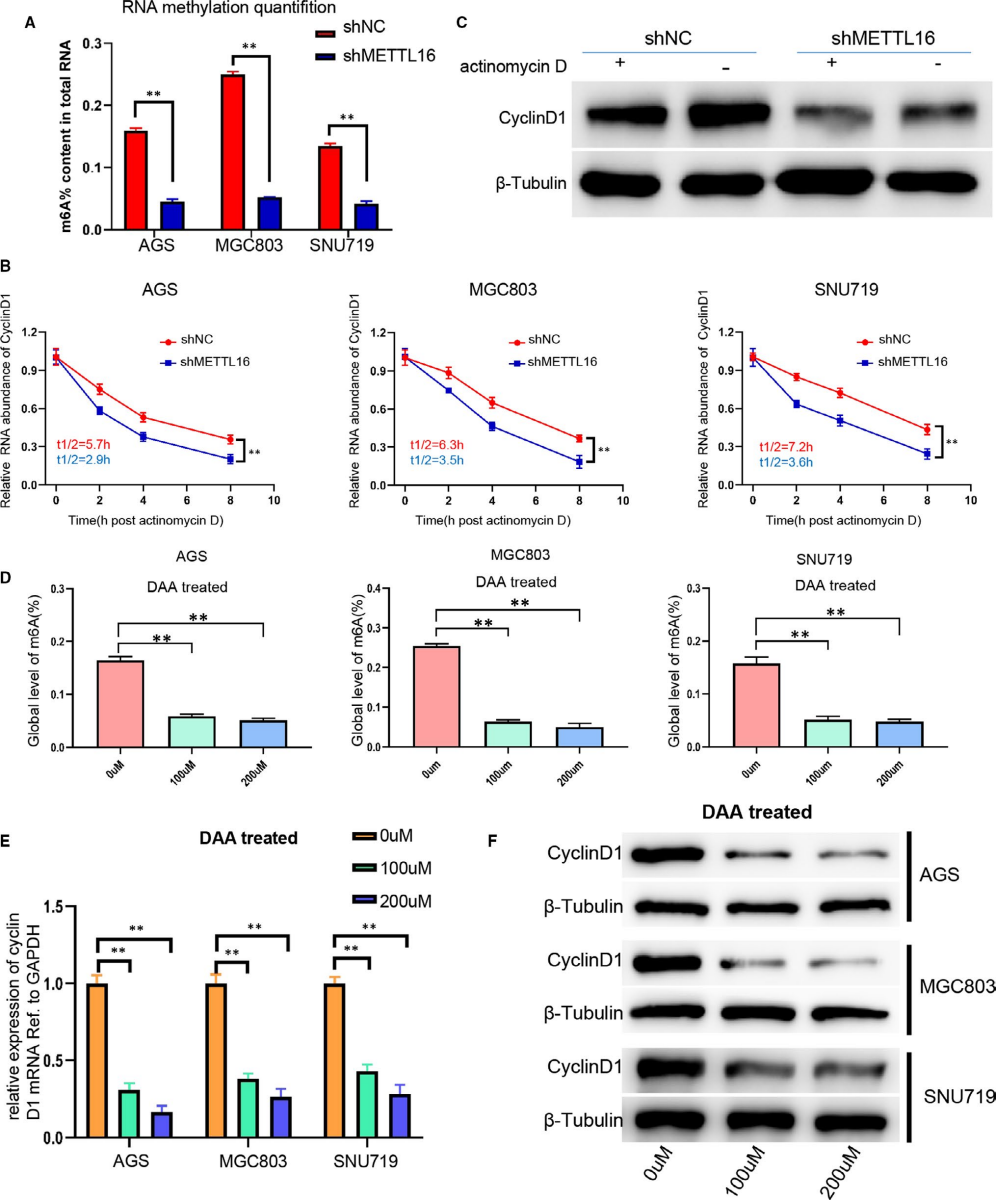
METTL16 regulates cyclin D1 expression via mediating the stability of cyclin D1 mRNA. (A) Based on the standard curve, RNA methylation quantitative analysis method was used to detect the overall content of m6A in shNC‐ or shMETTL16‐treated GC cells. (B) Cyclin D1 expression in shNC‐ or shMETTL16‐treated GC cells induced by actinomycin D for 8 h. (C) The RNA degradation rate was determined in shNC‐ or shMETTL16‐treated GC cells (referring to 0h). (D) The global level of m6A methylation in GC cells treated with different concentrations of DAA (0 umol/L, 100 umol/L, 200 umol/L) for 24 h. (E) The mRNA level of cyclin D1 in AGS, MGC803 and SNU719 cells treated with different concentrations of DAA (0 umol/L, 100 umol/L, 200 umol/L) for 24 h. (F) Western blotting analysis was performed to detect cyclin D1 expression in AGS, MGC803, SNU719 cells treated with different concentrations of DAA (0 umol/L, 100 umol/L, 200 umol/L) for 24 h

The above results have revealed that METTL16 could regulate cyclin D1 expression. It is known that METTL16 is an RNA methyltransferase which can methylate RNA; we further studied that whether METTL16 can regulate the expression of cyclin D1 through mediating the methylation of cyclin D1 mRNA. Firstly, we detected the m6A modification function of METTL16 and found that the total m6A level in GC cells was significantly reduced after knocking down METTL16 (Figure 7A). Subsequently, we conducted RNA degradation assays and found that the degradation rate of cyclin D1 mRNA in METTL16 knocked down GC cells was significantly faster than that in normal control group after treated with actinomycin D, meaning that the half‐life of cyclin D1 mRNA was significantly shortened (Figure 7B). Western blotting results also showed that the cyclin D1 expression was decreased in METTL16 knocked down GC cells with treatment of actinomycin D for 8 hours (Figure 7C). These results showed that METTL16 can promote cyclin D1 mRNA stability in GC cells, thereby promoting the expression of cyclin D1. Besides, we used methyltransferase inhibitors to treat GC cells (AGS, MGC803 and SNU719) and found that the m6A levels were reduced (Figure 7D), indicating that METTL16  functions as a RNA methyltransferase to regulate the cyclin D1 expression in GC cells.

Then, we carried out salvage experiments with AGS and MKN28. First of all, we treated lentivirus‐OE METTL16 or normal control cells with or without DAA and found that DAA could significantly inhibit the expression of cyclin D1, thus showing the regulation of cyclin D1 by METTL16 through its RNA methyltransferase activity (Figure S2). Similarly, with or without the addition of imperatorin, a specific inhibitor of cyclin D1, in OE and NC cells, CCK8 test, colony formation test and Edu test further proved that cyclin D1 is a downstream effector molecule of METTL16 (Figures S3 and S4).

In addition, methyltransferase inhibitor was used to study whether cyclin D1 expression is regulated by methyltransferase. The results showed that both mRNA and cyclin D1 protein levels were inhibited in GC cells after treated with DAA which is a methyltransferase inhibitor (Figure 7E,F), suggesting that methyltransferase plays a significant role in regulating the expression of cyclin D1 in GC cells. The above results suggested that METTL16 could enhance the stability of cyclin D1 mRNA in GC cells through m6A modification.

## 4 DISCUSSION

m6A RNA modification was first discovered in the 1970s^15^ and recently becoming a research hotspot in molecular regulation of epigenetics.^16^ Because m6A methylation plays an important role in regulating gene transcription and cell biology, it is inferred that m6A methylation is a key regulator in the process of human cancer. A growing body of evidence indicates that dysregulation of m6A modification plays significant roles in the pathogenesis of many different types of cancer.^17^ As a ‘writer’, m6A methyltransferase is undoubtedly an extremely important class of molecules in the process of m6A modification. So far, two kinds of RNA methyltransferases have been discovered, of which METTL16 is the second methyltransferase discovered in recent years, which can modify RNA with m6A,^9^ and has important physiological and biological functions. The role of METTL16 in cancer is currently researched mainly on hepatocellular carcinoma. Pei Wang et al believe that METTL16 gene deletion is an independent risk factor for DFS and indicates poor OS and DFS in liver cancer patients.^18^ However, the role of METTL16 in GC still eludes us.

In this study, we firstly detected both mRNA and protein levels of METTL16 in 16 pairs of GC tissues and its paired NATs along with 6 GC cell lines. We found that the expression of METTL16 in GC cells and tissues was significantly increased compared with the normal control group (Figure 1), consistent with the results of the query in the TCGA database. Further, the expression of METTL16 was negatively associated with OS in GC patients, similar with the results of METTL3 in GC.^19^ In contrast, elevated expression of METTL16 predicts higher OS in liver cancer patients.^18^ We believe that the difference of prognostic significance of METTL16 between GC and liver cancer is due to the fact that the same gene encoding the methyltransferase plays different roles in different cancers.^20^ For example, in the study of glioblastoma stem cells, researchers found that knocking out METTL3 could greatly promote the growth, self‐renewal and tumorigenesis of human glioblastoma stem cells.^21^ However, in a study of acute myeloid leukaemia, METTL3 was found to be necessary for the growth of acute myeloid leukaemia cells and down‐regulation of METTL3 could lead to cell cycle arrest, leukaemia cell differentiation, and even unable to induce leukaemia in immunodeficient mice.^22^, ^23^ This is why the research on m6A modification is unique, and the study we conducted is meaningful.

In this study, we found that METTL16 knock‐down could inhibit GC cell proliferation and tumour growth in mice, and the total m6A level of RNA was reduced in METTL16 knocked down or methyltransferase inhibitor–treated GC cells, including AGS, MGC803 and SNU719. In addition, it has been proved that knocking down METTL16 could lead to a reduction in the overall level of cellular RNA methylation.^24^ One previous study also showed a significant rise in the m6A methylation of total RNA in GC cells and tissues.^19^ These results indicated that METTL16 played a significant role in promoting cell proliferation by increasing the enzymatic activity of m6A in GC cells.

Additionally, we found that knock‐down of METTL16 induced significant arrest of GC cells in G1 phase. Then, we detected the key proteins which regulate G1 phase to S phase transitions in GC cells^25^ and found that the cyclin D1 expression was significantly reduced in METTL16 knocked down GC cells. Previous studies have shown high expressions of cyclin D1 in GC tissues, and cell cycle–related molecules are involved in the occurrence and progression of GC.^26^ It is also known that cyclin D1 and cyclin E are ‘restrictions’ for the transition from G1 to S, and cyclin D1, as a G1 phase cyclin locating in the nucleus, can accumulate and reach the highest level in the early and mid G1 phases.^27^, ^28^, ^29^ After cells pass through these two ‘restrictions’ and enter S phase, cyclin D1 and cyclin E will degrade or disappear in the nucleus. Inhibiting the function of cyclin D1 can prevent cells from entering the S phase from G1 phase, and cyclin D1 overexpression can compress the G1 phase of the cell cycle.^28^ Our results showed that GC cells were arrested in G1/S phase after knocking down METTL16, and both mRNA and protein levels of cyclin D1 were down‐regulated significantly. A similar phenomenon was also observed through immunofluorescence. Therefore, we concluded that knock‐down of METTL16 could arrest GC cell cycle through down‐regulating cyclin D1 expression.

METTL16 is currently considered to be an m6A RNA methyltransferase and a type of ‘writer’ independent of the METTL3/METTL14 complex and functions in mediating RNA stability,^30^ pre‐mRNA splicing^31^ and translation efficiency.^32^ Unlike METTL3 which tends to methylate RNA containing RRACH motif (R = A or G; H = A, C or U), the substrate catalysed by METTL16 contains the specific motif UACAGAGAA (methylated adenosine is underlined).^33^ Another difference from METTL3 is that METTL16 can methylate substrate RNA without binding to other proteins or components. However, METTL3 must form a methyltransferase complex with METTL14, WTAP, VIRMA, RBM15 and ZC3H13 to methylate the substrate RNA.^31^ Currently known substrates of METTL16 are U6 snRNA,^34^ long‐chain non‐coding ribonucleic acid MALAT1 and XIST^9^, ^34^, ^35^ and MAT2A mRNA.^12^ Studies have also shown that METTL16 uses a mixture of structure and sequence to identify its RNA substrates, and METTL16 has an additional role in the pre‐mRNA splicing process, enabling METTL16 to be both the ‘writer’ of m6A and the ‘reader’ of m6A.^34^ We have confirmed that the half‐life of cyclin D1 mRNA was shortened and its stability was also significantly reduced in METTL16 knocked down GC cells compared with the control group. We also found that the mRNA and protein levels of cyclin D1 were inhibited after treatment with methyltransferase inhibitors. These results suggested that METTL16 enhanced the stability of cyclin D1 mRNA through its methyltransferase activity, thereby increasing cyclin D1 expression to promote the proliferation of GC cells.

However, we are not yet certain that cyclin D1 mRNA is the direct substrate of METTL16, nor is it clear whether METTL16 can splice the pre‐mRNA of cyclin D1 and promote translation of cyclin D1. If METTL16 acts as a ‘writer’ to modify cyclin D1 mRNA with m6A, whether there is a recognition protein or ‘reader’ and a demethylase or ‘eraser’ for subsequent processing, these questions should be answered with further research.

## 5 CONCLUSION

In summary, the present study reveals that METTL16 has a cancer‐promoting effect in GC and high expression of METTL16 indicates poor prognosis of GC. METTL16 functions as an m6A methyltransferase to promote GC cell proliferation through enhancing the stability of cyclin D1 mRNA (Figure S5). Our findings enrich the functions of m6A methylation in tumour markers and shed light to a potential way to explore effective strategies for the treatment of GC.

## CONFLICT OF INTERESTS

The authors declare that they have no conflict of interest.

## AUTHOR CONTRIBUTIONS

**xiaokun wang:** Data curation (lead); Formal analysis (equal); Methodology (equal); Writing‐original draft (lead); Writing‐review & editing (equal). **Yawei Zhang:** Data curation (equal); Formal analysis (equal); Methodology (equal); Software (equal); Supervision (equal); Validation (equal); Writing‐original draft (equal); Writing‐review & editing (equal). **Chunming Wang:** Conceptualization (equal); Data curation (equal); Formal analysis (equal). **Bo Li:** Data curation (equal); Investigation (equal). **Tianzhi Zhang:** Data curation (supporting); Formal analysis (supporting); Software (supporting). **Wenjie Zhou:** Data curation (supporting); Investigation (supporting); Software (supporting). **Lyu‐jia Cheng:** Investigation (supporting); Software (supporting); Writing‐original draft (supporting). **Mingyu Huo:** Conceptualization (equal); Methodology (equal); Validation (equal); Writing‐original draft (equal); Writing‐review & editing (lead). **Changhua Zhang:** Project administration (equal); Resources (lead); Supervision (lead); Writing‐review & editing (equal). **Yulong He:** Funding acquisition (lead); Project administration (lead); Resources (lead); Supervision (lead); Writing‐review & editing (equal).

## ETHICS STATEMENT

Ethical committees of the First Affiliated Hospital of Sun Yat‐sen University and the Seventh Affiliated Hospital of Sun Yat‐sen University approved this study. Written consent was obtained from all individual participants included in the study.

## CONSENT FOR PUBLICATION

Not applicable.

## Supporting information

Fig S1

Fig S2

Fig S3

Fig S4

Fig S5

## Data Availability

The data that support the findings of this study are available from the corresponding author upon reasonable request.
