# Supplementary figures and images for: METTL16 promotes cell proliferation by up‐regulating cyclin D1 expression in gastric cancer
Source: J Cell Mol Med. 2021 Jun 2;25(14):6602–17. doi: 10.1111/jcmm.16664 (PMC8278090; doi:10.1111/jcmm.16664)

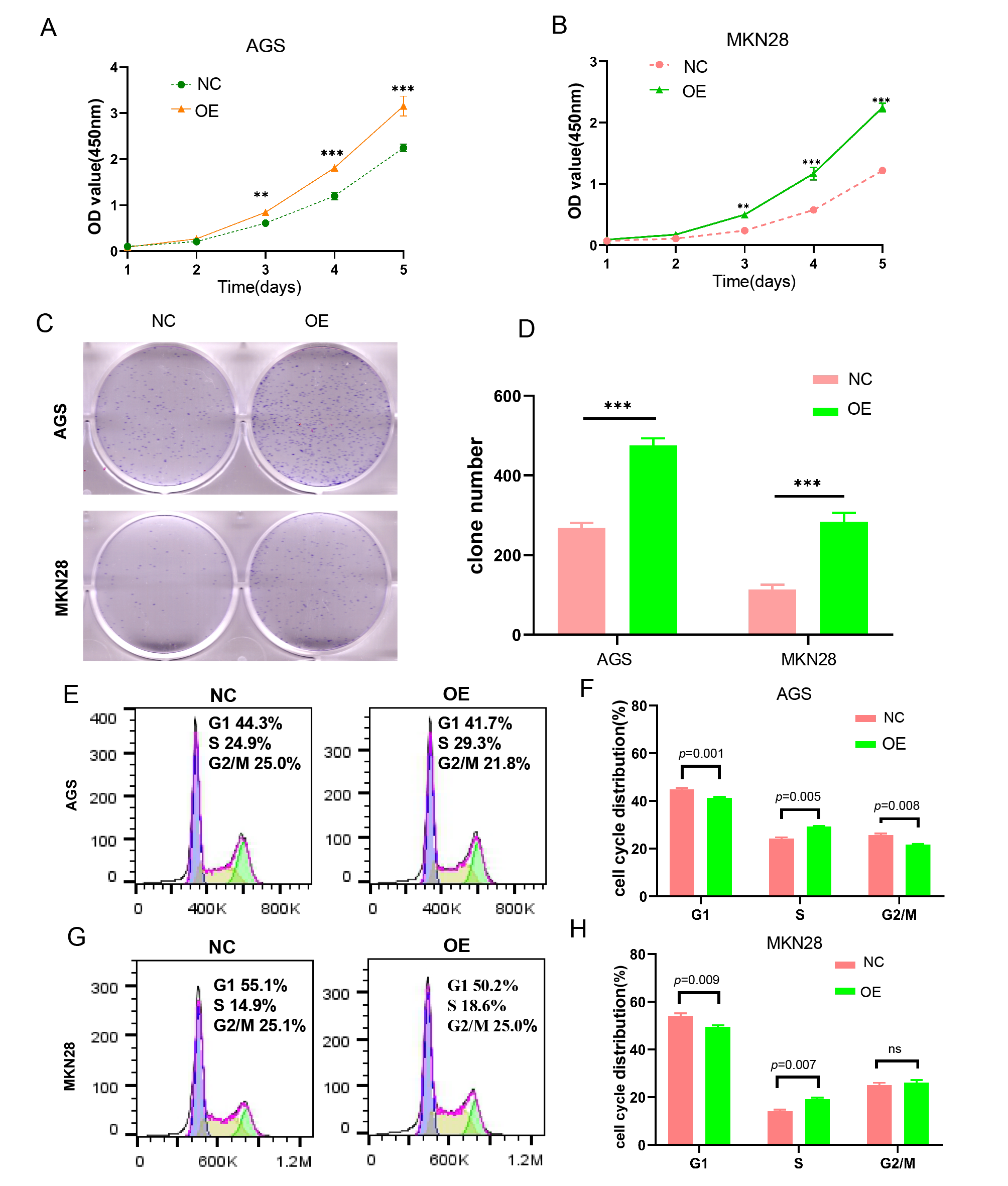

Supplement: Supplementary file 1 — Fig S1 [file JCMM-25-6602-s004.tif]

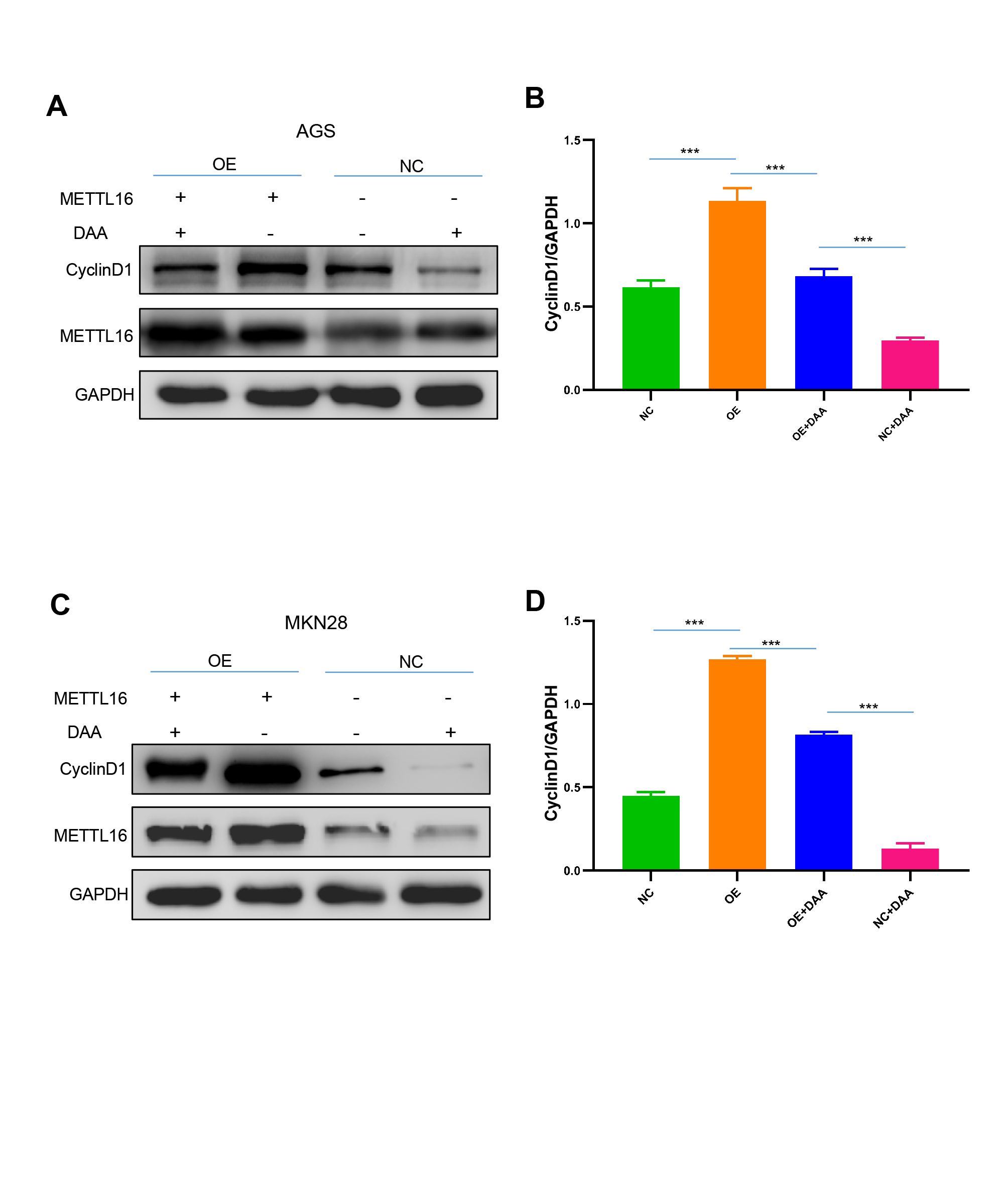

Supplement: Supplementary file 2 — Fig S2 [file JCMM-25-6602-s003.tif]

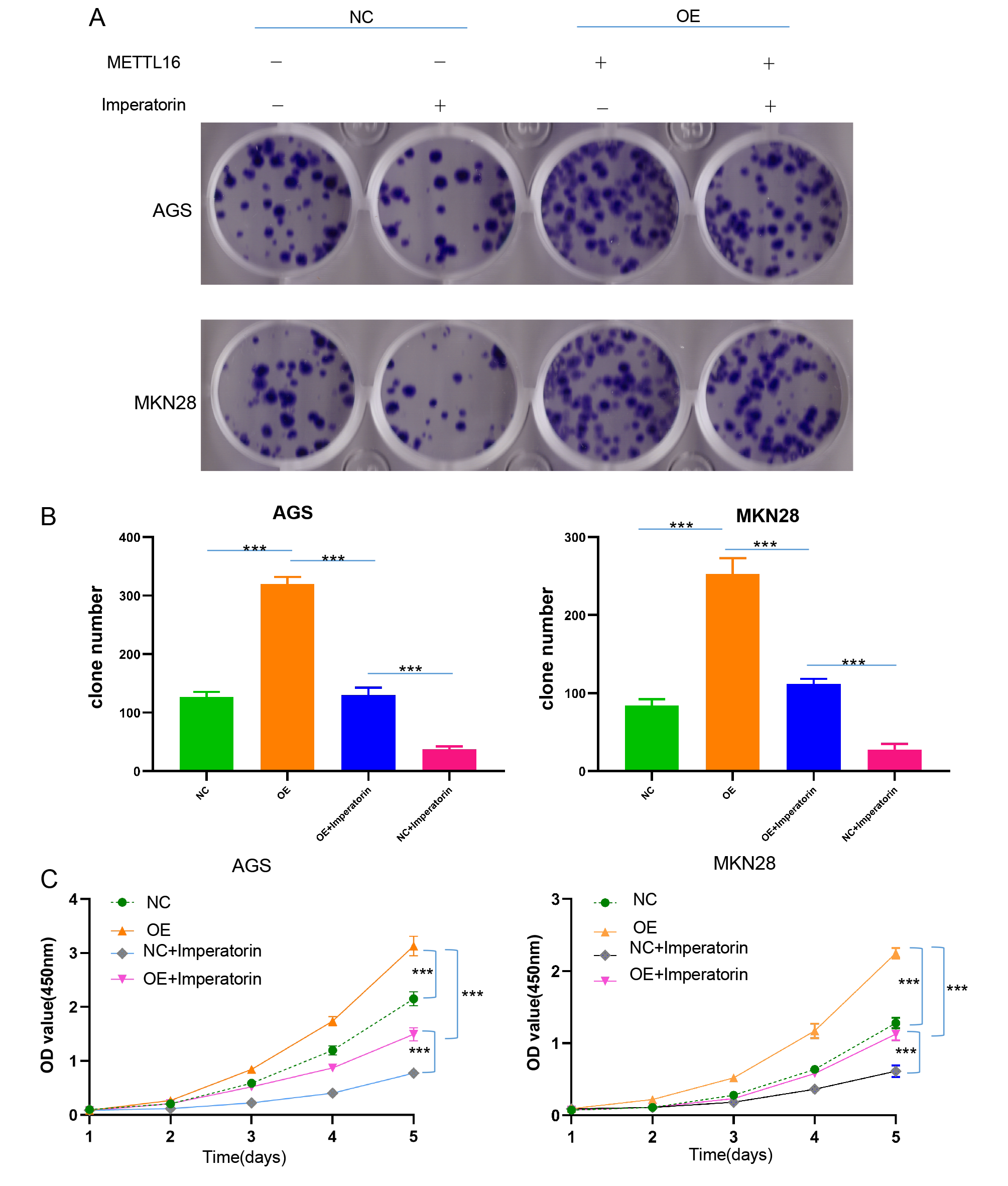

Supplement: Supplementary file 3 — Fig S3 [file JCMM-25-6602-s001.tif]

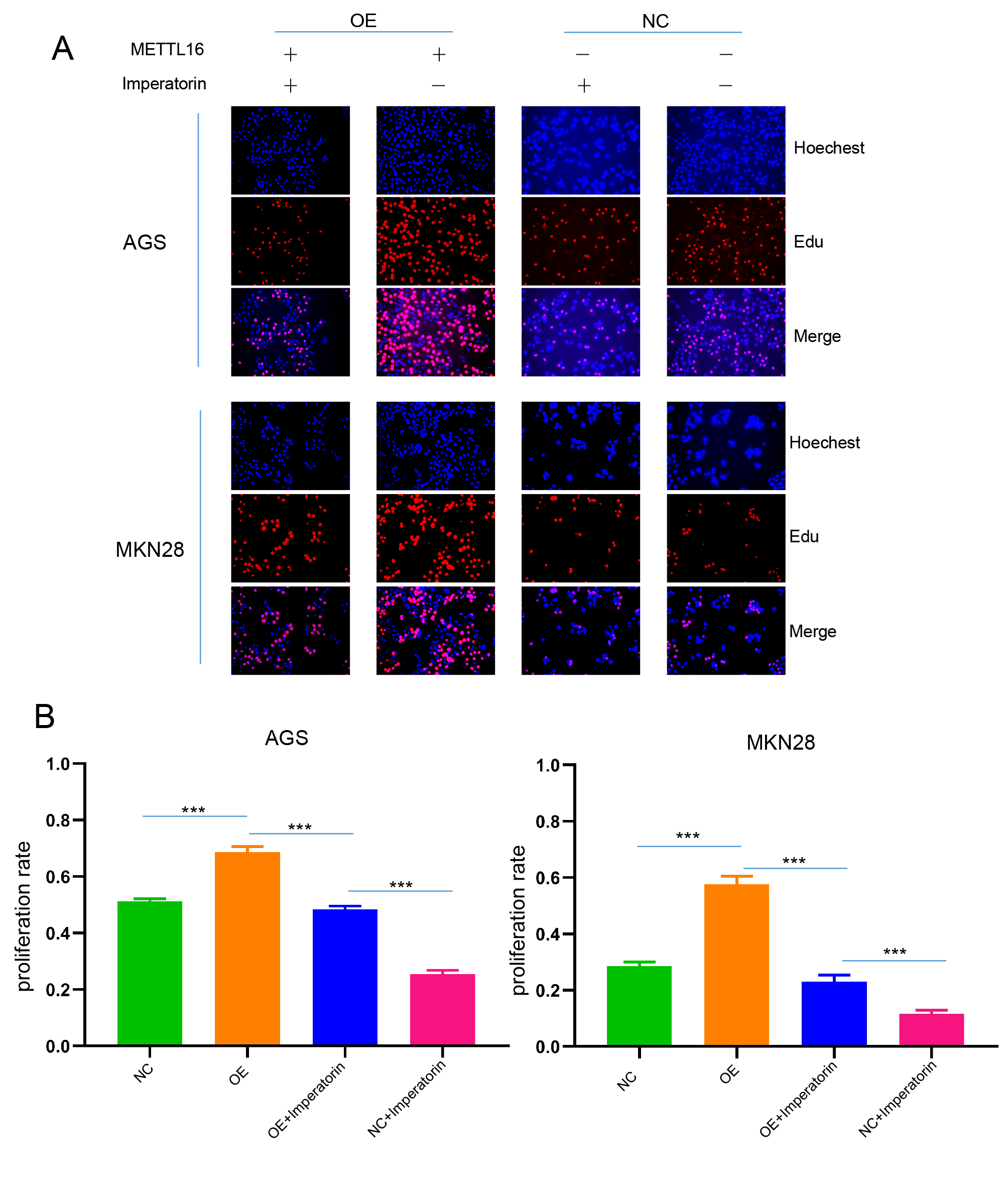

Supplement: Supplementary file 4 — Fig S4 [file JCMM-25-6602-s002.tif]

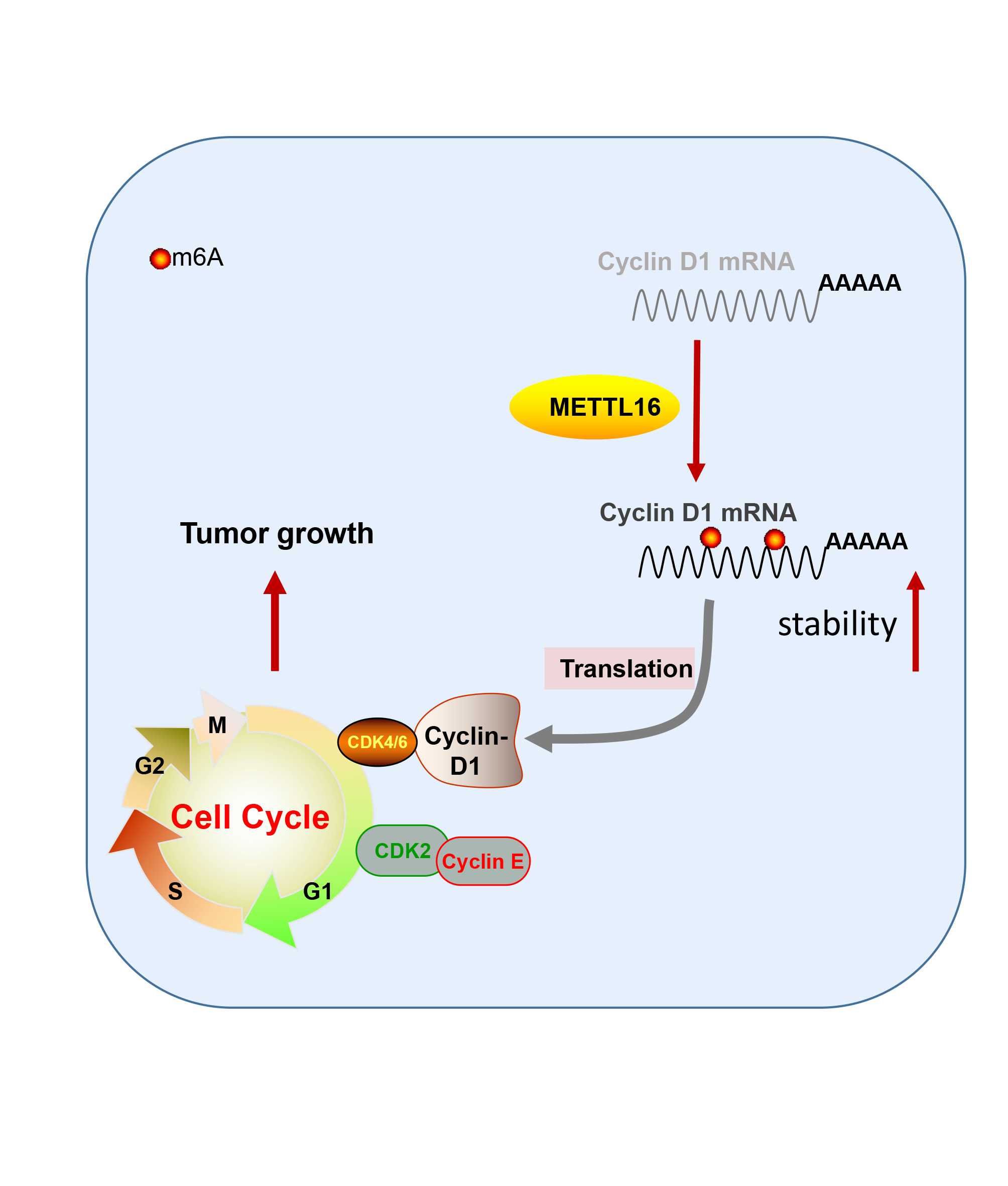

Supplement: Supplementary file 5 — Fig S5 [file JCMM-25-6602-s005.tif]
